# Targeted ANP32E Mutant Mice Do Not Demonstrate Obvious Movement Defects

**DOI:** 10.1371/journal.pone.0063815

**Published:** 2013-05-13

**Authors:** Peiyan Wong, Vonny I. Leo, Meijun Low, Tak W. Mak, Xiaodong Zhang, Patrick T. Reilly

**Affiliations:** 1 Neuroscience and Behavioral Disorders Program, Duke-NUS Graduate Medical School, Singapore, Singapore; 2 Laboratory of Inflammation Biology, Division of Cellular and Molecular Research, National Cancer Centre Singapore, Singapore, Singapore; 3 The Campbell Family Institute for Breast Cancer Research, University Health Network, Toronto, Ontario, Canada; 4 Department of Physiology, National University of Singapore, Singapore, Singapore; 5 Department of Psychiatry and Behavioral Sciences, Duke University Medical Center, Durham, North Carolina, United States of America; University of Florida, United States of America

## Abstract

**Background:**

The ANP32 family of proteins have been implicated in neuronal function through biochemical and cellular biology studies in neurons, as well as by recent behavioural studies of a gene-trapped loss-of-function mutation of *Anp32e* in mice, particularly with respect to fine motor function. A second targeted allele of the *Anp32e*, however, did not appear to demonstrate neurological phenotypes.

**Methodology/Principal Findings:**

Using a stringently controlled cohort of ten-generation backcrossed, co-caged, sex-matched, littermate pairs, we assayed for potential motor defects in the targeted ANP32E-deficient mice. We found no phenotypic difference in any assays.

**Conclusion:**

Since it is unlikely that the gene-trap is a more complete loss-of-function, our results suggest that ANP32E has no appreciable effect on motor functions and that genetic background differences most likely account for the gene-trap phenomena.

## Introduction

The acidic (leucine-rich) nuclear phosphoprotein 32 kD (ANP32) family of proteins have been implicated in regulating neuronal function from several lines of inquiry. Firstly, the founding member of the family ANP32A (a.k.a. PHAPI, LANP, I1PP2A, pp32) was found to bind and colocalize with the spinocellular ataxia protein, SCA1 [1]. Secondly, the same protein was shown to affect phosphorylation of the microtubule-associated factor tau, which is abundant in neurons and notable in the pathogenesis of Alzheimer's disease [2], [3]. Finally, a separate family member, ANP32E (a.k.a. CPD1, LANP-L), was originally cloned from postnatal rat cerebellums [4] and is proposed to regulate synaptogenesis and Purkinje cell function [5].

Three of the annotated ANP32 genes, *ANP32A*, *ANP32B* and *ANP32E*, are expressed and are distinguished by the presence of two functional regions; an N-terminal leucine-rich domain and a C-terminal tail comprised predominantly of acidic amino-acid residues [6], [7]. These proteins have been ascribed a surprisingly diverse number of biochemical activities including inhibition of PP2A [8], [9], association with microtubules [3], [10], [11], apoptotic caspase inhibition [12]–[15], regulation of mRNA transport and stability [16]–[18], and control of gene transcription [19]–[24]. Whereas PP2A inhibition is most frequently reported as critical in ANP32-mediated neuronal regulation [2], [5], the regulation of transcriptional activity, potentially through E4F1, has also been suggested to mediate ANP32 neuronal effects [25], [26].

Recently, two loss-of-function mutant alleles of *Anp32e* were characterized in mice. Mice homozygous for the targeted allele demonstrated no motor deficiency in qualitative analysis or in an accelerating rotorod analysis [27]. The “gene-trapped” allele carrying a “β-Geo” promoter trap insertion in intron 3, on the other hand, was analysed more thoroughly for movement disorders and was likewise normal in accelerating rotorod behaviour but had subtle phenotypes in balance beam and limb-clasp testing [28].

In an attempt to resolve the discrepancy between the results for the two lines of mice, we re-examined the targeted *Anp32e* allele that has been backcrossed ten generations into the C57BL/6 strain for a panel of movement assessments. Here we present the lack of evidence for any movement defects in the ANP32E-deficient mouse in balance beam performance or limb clasping. Similarly, we did not detect any gait, accelerating rotorod, and grip strength deficiencies in quantitative analyses. Since it is highly unlikely that this targeted allele retains more gene function than the trapped allele, we propose that previous findings are more likely due to genetic background of the mice used for the gene-trapped allele. Importantly, this study finds no detectable motor defects associated with ANP32E deficiency.

## Materials and Methods

### Mice

Targeted *Anp32e* heterozygous mice (*Anp32e* ^+/−^) were backcrossed an addition four generations into C57BL/6 background to give a final 10-generation C57BL/6 congenic. After final backcrossing, *Anp32e* ^+/−^ mice were interbred to give sex-matched littermate pairs of *Anp32e* nullizygous (*Anp32e* ^−/−^) and wild-type (*Anp32e* ^+/+^) mice. Genotyping was performed as previously reported [29]. All littermates were co-caged and provided 5% irradiated food and water *ad libitum*.

### Rotorod test

Mice were placed on the Rotor-rod apparatus (San Diego Instruments) that linearly accelerates from 4 to 40 rpm at a rate of 0.1 rpm/sec. Mice were tested in four trials over four consecutive days, with a 15 min rest period between trials. The latency to fall and distance travelled by a mouse were recorded.

### Balance beam test

Beams 50 mm in length, with two different widths, 6 and 12 mm, were used in the balance beam test for motor coordination [30]. A bright light was used as an aversive stimulus at the start platform and an escape box (20×20×20 cm) was placed at the end of the beam. The mice were trained for three trials on each beam for three consecutive days, and then tested on five consecutive days. The time taken to traverse the beam was recorded for each trial. The maximum time cutoff was 20 s. On the test days 1 to 5, the number of slips and grips were also recorded.

### Hindlimb clasping test

Mice were held by their tails and recorded for 30 s to measure for limb clasping behavior. Clasping behaviour was scored by an observer blinded to the individual mouse genotype.

### Runway test

Mice were allowed to free walk across a runway (Cleversys Inc.) from a white acrylic start box to a darkened escape box. The walls of the runway are 12 cm apart and its length is 100 cm, with a 0.8 cm thick glass floor. Adjustable light panels distribute light uniformly over the entire glass floor to enable the detection of the paws. Low intensity green background light allows for the separation of the body of the mouse from its brightly lit paws. Images are captured by a high-speed digital video camera mounted to record the ventral view of the runway (Basler Cam Inc.).

### Treadmill test

The treadmill (Columbus instruments, OH) was used for a forced walking gait analysis. The apparatus consisted of a motor-driven transparent belt with a 17 cm×5 cm compartment over it, where the mouse is placed. A high-speed digital video camera mounted to record the ventral view of the treadmill (Basler Cam Inc.).

### Automated gait analysis

Data acquisition and analysis of the gait in the runway and treadmill were done using Gaitscan (Cleversys Inc.). The training and analysis parameters have been described in detail elsewhere [31]. In brief, Gaitscan could reliably identify each paw and the measures taken include the stance, swing and stride times.

### Grip Strength test

Grip strength tests were performed using a grip strength meter from Columbus Instruments (Columbus, OH). The forelimb and full body grips of each mouse were measure in three successive trials and recorded. Hindlimb measures were calculated using the difference between the grams-force (gF) recorded for the full body and the forelimb. The results of the three tests were averaged for each mouse.

### Statistical analysis

Data were analyzed using R statistical package (R Foundation for Statistical Computing). Data are presented as means±SEM. Rotorod and balance beam data were analyzed using a mixed factorial design ANOVA. Between subjects factor for all tests was genotype, and within subjects factor for the rotorod and balance beam is test days. Measures scored in the hind limb clasping, runway and treadmill tests were analyzed using the two-tailed, paired t test. Bonferroni corrected pair-wise comparisons were used as the *post-hoc* tests. A *p*<0.05 was considered as significant.

### Ethics Statement

Mice were maintained under protocols 2009/SHS/447 and 2012/SHS/725, which were approved under the legal authority of the Singhealth Institutional Animal Care and Use Committee as per the Singapore N.A.C.L.A.R. guidelines. To alleviate undue suffering, all animals were socially housed in enriched environments with food and water available *ad libitum*. Animals were sacrificed either by carbon dioxide asphyxiation followed by cervical dislocation or by ketamine/medetomidine overdose followed by exsanguination.

## Results

In order to resolve the discrepancy between earlier publications regarding the phenotype of the ANP32E-deficient mouse, we generated seven co-caged, sex-matched, littermate pairs of *Anp32e* ^+/+^ and *Anp32e* ^−/−^ mice (Figure S1). Consistent with previous results in mixed-bred populations [27], [28], the C57BL/6-congenic *Anp32e* deficiency segregated at normal Mendelian ratios (Table S1). At ages between 16–20 weeks old, we performed an array of established motor tests.

We first examined whether the results of rotorod analysis from earlier cohorts in both publications consistently showed no effect in these mice. The seven pairs of mice were tested on an accelerating rotorod for four consecutive days, and the latency to fall was recorded. A mixed-design ANOVA revealed significant main effect of day (F_3,48_ = 24.3, p<0.0001), with no significant main effect of genotype and no significant interaction (Figure S2). Bonferroni corrected pair-wise comparisons for each test day showed no significant differences between genotypes, with both *Anp32e* ^+/+^ and *Anp32e* ^−/−^ mice showing improvement in performance with each increasing test day.

**Figure 1:**
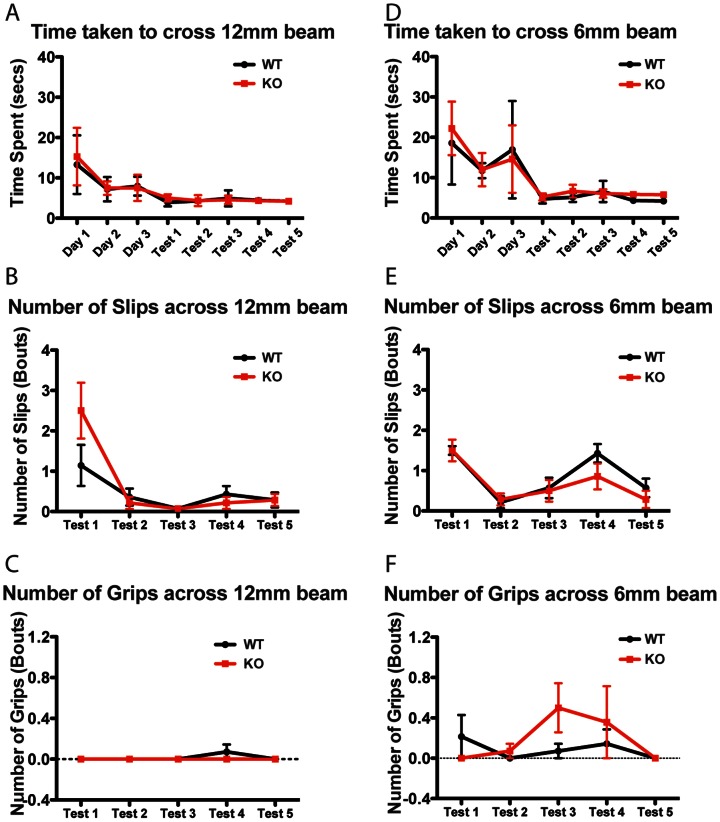
ANP32E-deficient mice perform normally on balance beam assay. Mice were assayed, on light aversion balance beam across a 12 mm diameter dowel (panels A–C) and a 6 mm diameter dowel (panels D–F), for crossing time (panel A and D), number of slips (panels B and E) and number of grips (panels C and F). No statistically significant differences were detected between *Anp32e* ^−/−^ (KO, red) and *Anp32e* ^+/+^ (WT, black).

Because the gene-trapped ANP32E-deficient mice have been reported to have poorer performance in balance beam testing, we assessed the motor balance and coordination abilities of the mice using the balance beam test. The time taken to cross were recorded for the 12 mm and 6 mm beams for the 3 training days and 5 test days. The mixed-design ANOVA only showed significant effects of test day for either beam (F_12,84_ = 21.3, p<0.0001 and F = 19.0_12,84_, p<0.0001 respectively), with no significant main effect of genotype and no significant interaction. For a more detailed analysis, the number of slips and grips were scored on the test days. The number of slips on the 12 or 6 mm beams showed significant main effects of only test day (F = 11.4_12,48_, p<0.0001 and F = 10.9_12,48_, p<0.0001 respectively). There were no significant main effects for the number of grips on the 12 or 6 mm beams. These results suggest that both *Anp32e* ^+/+^ and *Anp32e* ^−/−^ mice show improvement in the balance beam task across days, taking progressively shorter amounts of time to transverse the beams with fewer slips. Importantly, we saw no significant differences between genotypes (Figure 1).

The gene-trapped ANP32E-deficient mice have also been reported to have limb-clasping defects that are commonly associated with neuronal dysfunction. We, therefore, examined clasping response for the *Anp32e* ^+/+^ and *Anp32e* ^−/−^ littermates while being suspended from their tails for a total of 30 s. Using a variety of published quantification strategies [28], [32]–[34], we found no statistically significant differences between genotypes using any of the quantification methods. Indeed, a clasping response was only detected in one mutant mouse for a total duration of 1 sec in this first analysis (Table 1). Even using the most liberal clasping quantification measure we could find in the literature [35], where limbs are assessed independently and retraction was scored in the absence of clasping, we found only three mice generated any clasping score (1 point each of a possible 3) and each of those mice were wild-type, not the mutant (Table 1).

**Table 1:** Limb clasping assay quantification for targeted ANP32E-deficiency.

Genotype	Kular et al. Scoring [28] (average ± SEM)	Tanaka et al. Scoring [34] (average ± SEM)	Homma et al. Scoring [32] (average clasping time in sec ± SEM)	SHIRPA scoring [33], [36] (binary response)	Guyenet et al. Scoring [35] (average ± SEM)
Trial 1					
*Anp32e* ^+/+^	0±0	0±0	0 s±0 s	0 of 7	0.428±0.202
*Anp32e* ^−/−^	0.142±0.142	0.142±0.142	0.142 s±0.142 s	1 of 7	0±0
Trial 2					
*Anp32e* ^+/+^	0±0	0±0	0 s±0 s	0 of 7	0±0
*Anp32e* ^−/−^	0±0	0±0	0 s±0 s	0 of 7	0±0

Data shown are post-hoc analysis of hindlimb clasping tests for genotype-paired mice done in two trials on sequential days.

To confirm this result, we performed the assay again on the following day. In this analysis, we found no clasping response in the mice by any standard (Video SV1). Thus, we conclude that there is no apparent limb-clasping defect in the targeted ANP32E-deficient mouse.

**Figure 2:**
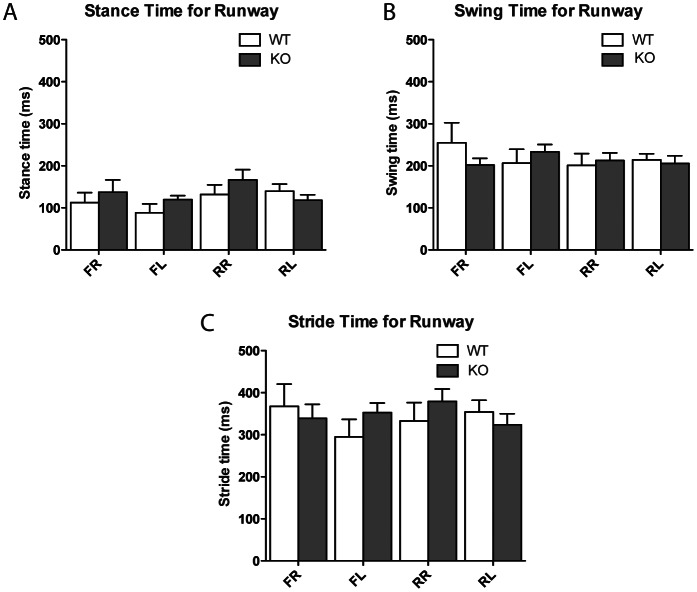
No phenotypic difference of ANP32E-deficient mice in free stride gait. Mouse gait analysis was performed on stationary runway and analysed for stance time (panel A), swing time (panel B) and stride time (panel C). Values for separate legs are presented separately. No statistically significant differences were detected by paired t-test analysis. *FR*, front right; *FL*, front left; *RR*, rear right; *RL*, rear right.

**Figure 3:**
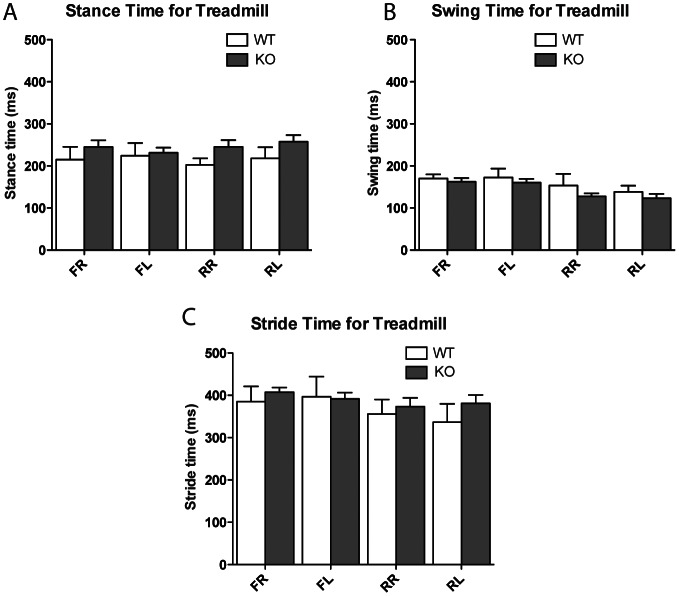
No phenotypic difference of ANP32E-deficient mice in forced stride gait. Mouse gait analysis was performed on a treadmill and analysed for stance time (panel A), swing time (panel B) and stride time (panel C). Values for separate legs are presented separately. *FR*, front right; *FL*, front left; *RR*, rear right; *RL*, rear right.

In order to further examine any potential motor defects in the targeted ANP32E-deficient mice, we performed gait analysis on these mice. Gait parameters such as swing, stance and stride time were measured in the runway (free-walk, Figure 2) and treadmill (forced-walk, Figure 3) gait tests. In neither of these gait tests did the gait measures for the front left, front right, rear left and rear right paws show any significant genotype differences, with the exception of the swing time for the rear left foot in the treadmill (*p* = 0.021). These results suggest that there were no major differences between *Anp32e* ^+/+^ and *Anp32e* ^−/−^ mice in both the runway and treadmill tests.

**Figure 4:**
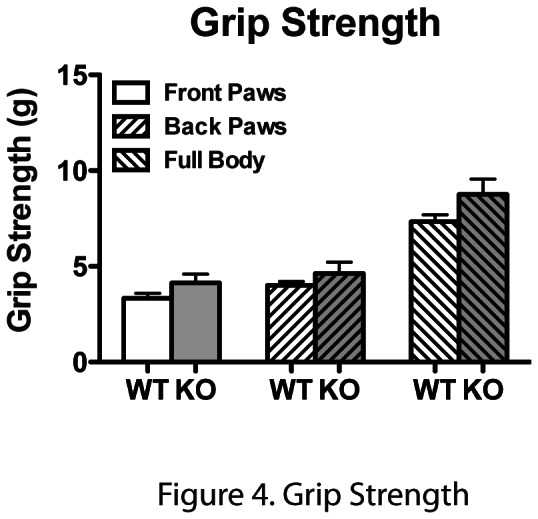
ANP32E-deficient mice do not show defects in grip strength. Mice were analysed for grip strength of front paws, hind paws, and total grip strength. No statistically significant differences were noted by paired t-test analysis.

Finally, in order to assess the neuromuscular function of the forelimbs, hindlimbs and whole body, we performed grip strength testing on these littermate pairs of *Anp32e* ^+/+^ and *Anp32e* ^−/−^ mice (Figure 4). The maximum force of pull for each test was recorded and normalized to the weight of each mouse. We could detect no statistically significant differences between the genotypes in these three tests.

## Discussion

ANP32E has been implicated in Purkinje cell function [5] and a previous report suggested subtle neurological defects with respect to motor function in a gene-trapped, *Anp32e*-deficient mutant [28]. In contrast, our previous limited analysis of motor function in the targeted *Anp32e* mutant mice did not show any phenotype [27]. To readdress in a more complete and quantitative manner, we performed a panel of motor-function assays on a stringently controlled cohort of mice. We found in no case, including balance beam performance and limb clasping, was a phenotype evident as was described for the gene-trapped *Anp32e* mutant.

There is little consistency for quantification of limb-clasping phenomena in the literature [28], [32], [34]–[36]. To ensure that we did not miss any phenotype due to scoring, we applied five different scoring strategies, including the same one used for the gene-trapped allele. Even the most liberal scoring strategy, whereby hindlimbs are treated individually and retraction without clasping is scored, did not give any statistically significant difference between the genotypes. Intriguingly, when we applied these scoring systems for the representative mutant video provided as an example in the gene-trapped analysis, we also did not score any limb-clasping positivity by these techniques. Indeed, we perceive the animal in this video attempting escape behaviour by including flexing of their trunks, which is generally not standard limb-clasping behaviour as described in the literature [35], [37].

Different alleles of the same gene can have different phenotypes and gene traps are known to previously give rise to hypomorphic or gain-of-function alleles. In the case of *Anp32e* however, both alleles purport to be complete loss-of-function alleles. Since gene-trap alleles depend on engineered splicing events to generate loss of function, which may not happen to completion, the targeted allele generated by removal of exons (four of six coding exons for *Anp32e*), therefore, is more likely to be a complete loss of function. Hence the difference in apparent phenotypes is unlikely to be due to more complete loss-of-function of the gene-trapped allele.

We have performed our experiments on a cohort of co-caged, sex-matched, littermate pairs from parents that were ten-times backcrossed into C57BL/6. We feel that this is critical for assessing subtle phenotypes, particularly where a single ES isolate is used. No mention is made of such refinements in analysis of the gene-trapped allele. In particular, the background differences could explain the differences in results. Significant strain-dependent differences have been noted in the Mouse Phenome Database for both balance beam performance and limb clasping response [34], [37]. The C57BL/6 performed much better than other strains although the 129P2/OlaHsd strain, the background of the gene-trapped allele, was not directly compared. Thus, to conclude that a mutation is responsible for a subtle effect, we propose it is critical to use a minimal of six-generations congenic.

It has been shown that rodent environment is critical for proper experimental control [38]–[40]. Our standard of using co-caged, littermate pairs provides maximum control of environmental factors that can influence behavioural tasks. The unequal numbers of different genotypes analysed for the gene-trap allele suggest that this cocaging of compared animals was likely not performed.

ANP32E is a highly conserved factor in mammals with expression in a broad array of tissues including neurons. In contrast to the conclusions of a previous report, we do not find any neurological phenotypes associated with the complete mutation of this gene. We propose that difference previously reported for the gene-trapped allele are likely due to linked genes on mouse chromosome 3 that will vary significantly in a mixed-bred background.

## Supporting Information

Figure S1 **PCR-based genotyping of the experimental cohort.** Ethidium bromide stained agarose gels of PCR reactions for sex-matched littermate pairs used in this analysis. The upper band of 850 bp detects *Anp32e* ^+^ (WT) allele whereas the lower band detects the *Anp32e* ^−^ (KO) band. *+/+*, mice ascertained to be *Anp32e* ^+/+^; *−/−*, mice ascertained to be *Anp32e* ^−/−^; *+/−*, mice ascertained to be *Anp32e* ^+/−^.
(EPS)

Figure S2 **No rotorod defect seen in the targeted ANP32E-deficient mouse.** Mice were analysed using the accelerating rotorod to determine latency time to fall (panel A) and total distance travelled (Panel B). No statistically significant differences were detected between *Anp32e* ^−/−^ (KO, red) and *Anp32e* ^+/+^ (WT, black).
(EPS)

Table S1 **Expected and observed Mendelian ratios of progeny derived from the intercrossing of C57BL/6 backcrossed Anp32e^+/−^ mice.** Data shown are the number of mice of a given genotype (percentage of total progeny). Chi square analysis determined that there was no significant difference from expected ratios.
(DOCX)

Video SV1 **No difference in limb-clasping behaviour in the mice.** Video presents recording of a representative littermate pair of mice second trial for limb-clasping assay. No limb-clasping or retraction is evident in either the *Anp32e* ^−/−^ (first animal shown) or *Anp32e* ^+/+^ (second animal shown).
(ZIP)
