# Supplementary material for: Targeted ANP32E Mutant Mice Do Not Demonstrate Obvious Movement Defects
Source: PLoS One. 2013 May 13;8(5):e63815. doi: 10.1371/journal.pone.0063815 (PMC3652840; doi:10.1371/journal.pone.0063815)
Supplement: Table S1 — Expected and observed Mendelian ratios of progeny derived from the intercrossing of C57BL/6 backcrossed Anp32e+/− mice. Data shown are the number of mice of a given genotype (percentage of total progeny). Chi square analysis determined that there was no significant difference from expected ratios. (DOCX) [file pone.0063815.s003.docx]

| \|  \| genotype \| \| \| \| --- \| --- \| --- \| --- \| \|  \| Anp32e^+/+^ \| Anp32e^+/-^ \| Anp32e^-/-^ \| \| expected \| 10.5 (25%) \| 21 (50%) \| 10.5 (25%) \| \| observed \| 10 (23.8%) \| 20 (47.6%) \| 12 (28.6%) \| |
| --- | --- | --- | --- | --- | --- | --- | --- | --- | --- | --- | --- | --- | --- | --- | --- | --- |

Supplemental Table S1. Expected and observed Mendelian ratios of progeny derived from the intercrossing of C57BL/6 backcrossed Anp32e^+/-^ mice. Data shown are the number of mice of a given genotype (percentage of total progeny). Chi square analysis determined that there was no significant difference from expected ratios.
